# The opioid system in endometriosis: implications for endometrial receptivity and reproductive outcomes

**DOI:** 10.3389/fendo.2026.1857083

**Published:** 2026-06-29

**Authors:** Estibaliz Olabarrieta, Lide Totorikaguena, Sara Alonso-Fernández, Ekaitz Agirregoitia, Naiara Agirregoitia

**Affiliations:** 1 Department of Pharmacology, Faculty of Medicine and Nursing, University of the Basque Country UPV/EHU, Leioa, Bizkaia, Spain; 2 Department of Physiology, Faculty of Medicine and Nursing, University of the Basque Country UPV/EHU, Leioa, Bizkaia, Spain; 3 Department of Pharmacology/Biotechnology, Faculty of Biomedical and Health Sciences, European University of Madrid, Madrid, Spain

**Keywords:** endometrial receptivity, endometriosis, infertility, inflammation, non-hormonal therapy, opioid system

## Abstract

Endometriosis, a chronic inflammatory disease affecting 10–15% of women of reproductive age, remains a leading cause of female subfertility. While current management strategies focus on surgical excision and hormonal suppression, these approaches often fail to address the underlying reproductive dysfunction or are incompatible with pregnancy. In this context, the opioid system emerges as a factor of interest, though research has traditionally focused on it almost exclusively as a target for pain management. This review synthesizes existing evidence to provide a novel perspective on how the opioid system regulates endometrial function and reproductive health. We discuss the presence and cyclical fluctuations of opioid receptors and peptides within the uterine environment, highlighting their influence on tissue remodeling, angiogenesis, and apoptosis—processes that are frequently dysregulated in endometriosis. Despite the scarcity of recent clinical studies, the integration of these “non-analgesic” opioid pathways suggests that they may be active participants in the pathogenesis of the disease rather than mere bystanders in pain signaling. By connecting classical opioid research with modern challenges in endometriosis-associated infertility, this work identifies critical knowledge gaps and potential non-hormonal targets. Understanding these pathways is essential for developing therapeutic strategies that manage chronic pain while safeguarding the reproductive health of these patients.

## 1 Introduction

Endometriosis is a chronic, estrogen-dependent inflammatory disease defined by the presence of endometrial-like tissue outside the uterine cavity. It affects approximately 10–15% of women of reproductive age and is characterized by pelvic pain, chronic fatigue, and significant subfertility (1, 2).

Although its etiology remains debated, retrograde menstruation is considered the most widely accepted mechanism for endometriosis (3). However, a dysregulated immune response and hormonal imbalances are considered necessary for the survival and implantation of ectopic tissue (4). To survive in ectopic environments, endometriotic cells exhibit cancer-like hallmarks, such as altered integrin expression, increased matrix metalloproteinase (MMP) activity (5), a robust angiogenic capacity, and evasion of apoptosis (6). These interconnected processes, regulated by a complex network of endocrine and immune factors, facilitate the proliferation and dissemination of lesions.

Emerging insights suggest that the opioid system may be involved in these pathogenic processes. To date, scientific attention has primarily focused on the role of this system in pain management and the challenges of opioid-based analgesia. The goal of this review is to synthesize evidence on the molecular mechanisms of endometriosis and evaluate how they may be driven by opioid signaling, demonstrating that this relationship extends far beyond analgesia.

For this mini-review, a comprehensive literature search was conducted across the PubMed, Scopus, and Web of Science databases, covering publications predominantly from the last 20 years. Search terms included “opioid system”, “endometriosis”, “inflammation”, “angiogenesis”, “apoptosis”, “infertility”, and “opioid receptor”. Notably, the initial search yielded a vast majority of results focused on the clinical use of opioid analgesics for pain management and the associated risks of opioid use disorder. Therefore, we refined our selection to identify studies that explored the endogenous opioid system as a biological modulator of tissue pathophysiology. Given the scarcity of direct clinical evidence, our strategy prioritized a mechanistic approach, integrating human data on endometriosis with both human and preclinical evidence on opioid signaling to identify convergent molecular pathways.

## 2 Molecular mechanisms of lesion progression in endometriosis

### 2.1 Immune dysregulation and inflammation

Endometriosis is characterized by a disrupted cellular immunity, which maintains a pro-inflammatory environment while facilitating immune evasion. Peritoneal macrophages in these patients exhibit reduced phagocytic capacity, which is linked to downregulated MMP-9 and CD36, and a shift toward an M2-polarized, immunosuppressive phenotype (7). This environment is fueled by a bidirectional feedback loop in which prostaglandin E2, stimulated by elevated cyclooxygenase-2 (COX-2) levels, enhances aromatase activity, leading to local hyperestrogenism that further suppresses natural killer (NK) cell cytotoxicity and promotes lesion survival. This highlights the complex interplay between immune dysfunction and hormonal dysregulation (8, 9).

### 2.2 Survival mechanisms: angiogenesis and apoptosis evasion

Ectopic lesions evade physiological clearance through a reduced apoptotic rate, specifically via diminished FAS expression (10). Simultaneously, they exploit the endometrium’s inherent angiogenic potential, which is exacerbated by high levels of vascular endothelial growth factor (VEGF) and pro-inflammatory cytokines (IL-1β, IL-8, and IL-6). This angiogenic switch is critical for the establishment and progression of lesions (11).

### 2.3 Molecular drivers of invasion

TGF-β acts as a master regulator in this context, promoting neoangiogenesis, reducing NK cell activity, and facilitating disruption of the mesothelial monolayer for cellular invasion (12). This invasive capacity is further supported by a systemic imbalance between MMP (or MMPs, notably MMP-1, -2, -7, and -9) and their inhibitors, tissue inhibitors of metalloproteinases (TIMPs). All of these pathways converge on Mitogen-Activated Protein Kinase (MAPK) signaling, which chronically upregulates COX-2, driving the pain and infertility hallmarks of the disease (9).

## 3 Endometriosis and associated infertility

The causal link between endometriosis and infertility is well-established, particularly in severe cases. In advanced disease, pelvic distortion caused by endometriosis can lead to diminished ovarian reserve and pelvic adhesions, which can impair oocyte release from the ovary, inhibit ovum pickup, or hinder ovum transport (13).Endocrine disorders associated with endometriosis can contribute to luteinized unruptured follicle syndrome, impaired folliculogenesis, luteal phase defects, and premature, delayed, or multiple LH surges (14). Abnormal uterine contractility can also affect gamete and embryo transport and implantation. Other endometriosis-related pathophysiological mechanisms that result in impaired fertilization, embryogenesis, and embryo implantation include oxidative stress and immune system dysregulation (15). In all cases, it is essential to manage and monitor the disease while always considering the specific and individualized reproductive circumstances of each patient (16, 17).

## 4 Current treatments and clinical limitations

Endometriosis management relies on hormonal suppression (GnRH agonists, progestogens, and contraceptives) or surgical excision. However, hormonal therapies are inherently incompatible with patients seeking immediate pregnancy, as they inhibit ovulation and alter endometrial receptivity (18, 19). While surgery and Assisted Reproductive Technologies (ART) remain the primary options for restoring fertility, chronic pain often persists due to centralized nociceptive processing and inflammatory sensitization processes (20).

Although they are not first-line therapies, opioids are frequently prescribed for uncontrolled or post-surgical pain associated with this condition (21). However, their long-term efficacy is limited, and the associated risk of addiction remains a significant concern; furthermore, the potential complex interplay between the opioid system and the underlying mechanisms of the pathology remains largely unexplored (22). This knowledge gap is particularly concerning for patients prioritizing conception because specific agents, such as codeine, have been shown to suppress ovarian steroidogenesis and folliculogenesis (23). Together with the evidence discussed below, these findings suggest that the opioid system, traditionally considered a target for pain relief, may exert a broader influence on the biological mechanisms of the disease. These possibilities must be approached with caution; on the one hand, there is currently insufficient information to establish definitive links; on the other hand, it must be considered that, beyond the well-known risk of addiction, inappropriate opioid modulation may paradoxically disrupt reproductive function and exacerbate disease progression. Consequently, there is an urgent need to elucidate these intricate interactions to determine whether the opioid system can be effectively harnessed as a non-hormonal therapeutic target without compromising reproductive health or patient safety. In the following sections, we will examine the role this pathway may play in reproductive physiology.

## 5 The opioid system as a modulator of reproduction

The endogenous opioid system comprises four G-protein coupled receptors: the mu (MOR), delta (DOR), kappa (KOR), and nociceptin-orphanin FQ peptide (NOP) opioid receptors. These receptors bind to specific ligands derived from four precursors: proopiomelanocortin (POMC), proenkephalin (PENK), prodynorphin (PDYN), and pro-nociceptin (pPNOC) (24).

This system is a key regulator of the reproductive axis; while opioids modulate gonadotropin-releasing hormone (GnRH) and luteinizing hormone (LH) secretion at the hypothalamic-pituitary level, they also exert direct autocrine and paracrine effects within the reproductive tract (25).

Components of the opioid system are widely expressed in the mammalian ovary, including follicles, granulosa cells, and oocytes (26–31). Their expression is dynamically modulated by follicle-stimulating hormone (FSH) and LH, which highlights their role in regulating folliculogenesis, oogenesis, and steroid hormone secretion (32). These interactions underscore the opioid system’s direct impact on fertility and endometrial function (**Table 1**).

**Table 1:** An overview of the implications of the opioid system in female reproduction.

Opioid system	Ovary	Oviduct	Uterus
Opioid receptors			
MOR	Present in human granulosa cells and oocytes (30). Morphine and naloxone influence oocyte maturation and fertilization of various species (31, 33–34, 86).	Present in cells of the mare oviduct (35).	Described in the human endometrium. ↑ its expression during the ovulation phase of the human menstrual cycle (36).
DOR	Present in the granulosa cells and oocytes of mammals, including humans (30).		Described in the human endometrium. ↑ its expression in the early secretory phase of the human menstrual cycle (37)
KOR	Present in the granulosa cells and oocytes of mammals, including humans (30, 38).		Described in the human endometrium. ↑ its expression in luminal epithelium from the early secretory phase of the human menstrual cycle (37)
Opioid precursors			
PDYN	Modulated by gonadotropins in porcine granulosa cells and theca cells (29).	Present in the oviduct of mice. Its expression changes during embryo development (39).	Described in human endometrial cells (40).
POMC	Present in the antral follicles, corpus luteum, granulosa, and theca cells in different species (26–29).	Present in the oviduct of mice. Its expression changes during embryo development (39).	Described in human endometrial cells (40).
PENK	Induced by LH in porcine theca cells (29).	Present in the oviduct of mice. Its expression changes during embryo development (39).	↑its expression close to the implantation site in mice (41, 42). Described in primates (43).
Opioid peptides			
β-endorphin	Present in the ovaries and follicular fluid of both bovine and murine species (28, 44). Modulates oocyte maturation and ovulation in women (45).	Present in bovine oviductal fluid but in lower concentrations (44).	↑ from the follicular phase to the luteal phase in humans (44). ↑ estrogen receptors (46).
Met-Enk	Present in bovine follicular fluid (44).	Present in bovine oviductal fluid, but in lower concentrations (44).	↑ from the follicular phase to the luteal phase in humans (44).

Evidence suggests that the opioid system plays a pivotal role in the preimplantation environment, particularly in facilitating successful oocyte transport through the oviduct. While opioid concentrations in oviductal fluid are lower than in other regions of the reproductive tract, their precursor profiles vary significantly throughout early development (44). Notably, exogenous morphine administration has been shown to impair normal embryo development in mice, evidencing the sensitivity of this phase to opioid modulation (39).

Within the uterus, the primary receptors (MOR, DOR, and KOR) are widely expressed across mammalian species (43, 46). Their regulation is highly hormone-dependent: in rodents, progesterone stimulates *PENK* transcript levels, whereas in primates, estrogen primarily regulates PENK expression in the endometrium, an effect antagonized by progesterone (47). In humans, POMC and PDYN precursors have been identified in endometrial cells, with PENK mRNA levels peaking specifically near the implantation site (40–42).

Consistent with these regulatory patterns, the human endometrium exhibits dynamic fluctuations of opioid peptides across the menstrual cycle. Concentrations of β-endorphin and Methionine-Enkephalin (Met-Enk) increase significantly from the follicular to the luteal phase (38, 44). Furthermore, β-endorphin, acting via MOR, enhances estrogen receptor expression, creating a feedback loop that prepares the tissue for receptivity (46). Our research group has confirmed that MOR, DOR, and KOR exhibit cyclical variations in the human endometrium, with MOR expression reaching a maximum during the LH surge and ovulation (36, 37).

Functionally, this system is postulated to regulate uterine physiology through several interconnected pathways:

-Uterine Remodeling: Opioids mediate apoptosis and facilitate embryo orientation prior to implantation (48, 49).
-Angiogenesis and Decidualization: The opioid system has been implicated in angiogenic processes across various tissues (50). Specifically, exogenous morphine administration in mice has been shown to impair luminal epithelial differentiation, decrease stromal cell proliferation, and lead to poor angiogenesis, collectively compromising uterine receptivity (51).
-Cell Proliferation: Opioid peptides act as negative regulators of uterine growth during ontogeny by interacting with epidermal growth factor (EGF) and estradiol, as observed in rat models (52, 53).
-Uterine Motility: In the uterine fluid of gilts and rats, β-endorphin has been shown to inhibit spontaneous contractions by modulating prostaglandin synthesis and calcium uptake, thereby ensuring a quiescent environment for the embryo (54, 55).

Taken together, these findings highlight the opioid system as a critical factor in reproductive success. Consequently, any pathological or exogenous disruption of this signaling can severely impair uterine receptivity, as exemplified by the previously mentioned effects of morphine during early gestation, during which reduced implantation sites and defective angiogenesis were observed (51). This evidence underscores the broader risk that opioid disruption poses to the structural and functional integrity of the reproductive environment. Consequently, increasing emphasis is being placed on the potential involvement of the opioid machinery in various endometrial pathologies. Remarkably, the physiological processes regulated by opioids—such as remodeling, angiogenesis, and cell proliferation—closely mirror the very mechanisms known to be altered in endometriosis. This suggests that this system’s malfunction could be a pivotal factor in endometriosis-associated subfertility. Therefore, further research is crucial to comprehensively characterize this system and explore its viability as a therapeutic target.

## 6 The opioid system involvement in endometriosis and associated infertility

Beyond the aforementioned biological overlap, clinical evidence has revealed altered opioid receptor expression profiles in patients with endometriosis, distinguishing them from healthy counterparts. At the clinical level, MOR expression has been shown to fluctuate in response to hormonal treatment (56). Moreover, studies have indicated that this receptor is upregulated in the endometrial stromal cells of affected women, particularly during the proliferative phase (57). This overexpression is linked to the over-activation of the MAPK and PI3K/AKT pathways, which are critical for the initial development of ectopic lesions (58). Conversely, some studies have reported lower plasma levels of β-endorphin and dynorphin in these individuals, suggesting a systemic opioid imbalance that correlates with severe dysmenorrhea (59).

### 6.1 Inflammation and immune modulation

Although there is limited research directly investigating the mechanisms underlying endometriosis-related symptoms and their potential opioid involvement, several connections can be hypothesized based on the established roles of opioids in various processes relevant to the disease. It is well-established that inflammation is a key process in endometriosis, and the opioid system plays a significant role in inflammatory responses. As reviewed by Iwaszkiewicz et al., the number of opioid receptors increases in peripheral tissues during inflammation (60). In the initial stages of inflammation, neutrophils serve as the primary opioid-containing leukocytes. Subsequently, macrophages and lymphocytes, including T cells, predominate at later stages, mirroring their characteristic order of infiltration into inflamed tissues. Notably, inflammation has been shown to increase the expression of opioid peptides in these immune cells. In fact, all major opioid peptides and their precursor mRNAs have been identified in immune cells, with β-endorphin being the most prevalent. It has been found that cytokines, such as IL-8, can stimulate the secretion of opioids from these cells (60). Met-Enk plays a significant role in both humoral and cell-mediated immune responses. Peripheral administration of Met-Enk in rats elicits a biphasic and dose-dependent immunomodulatory effect: high doses of Met-Enk suppress immune reactivity, while low doses potentiate it (61). In contrast, KOR-mediated responses suppress humoral immune responses (62). Along with all of these findings, opioids are also reactive oxygen species (ROS) producers, and they inhibit the function of enzymatic antioxidants (63), which are known to promote inflammation.

### 6.2 Mechanisms of survival (angiogenesis/apoptosis) and invasion

As previously mentioned, opioids are also able to mediate processes such as angiogenesis and apoptosis in the endometrium. However, the precise effects of opioid signaling on angiogenesis remain poorly defined, as the majority of available data stems from cancer models. Evidence suggests that MOR activation triggers angiogenic pathways (64, 65) while KOR agonists could suppress VEGF signaling, inhibiting this process (50). Nevertheless, this could also be a dose- and/or tissue-dependent issue (66). Studies have suggested that morphine’s pro-angiogenic effects, observed at clinically relevant concentrations, may be associated with tumor-promoting side effects, while KOR agonist Nalfurafine may enhance the anti-angiogenic therapeutic efficiency of the VEGF/VEGF-receptor targeting drugs (67).

**Figure 1:**
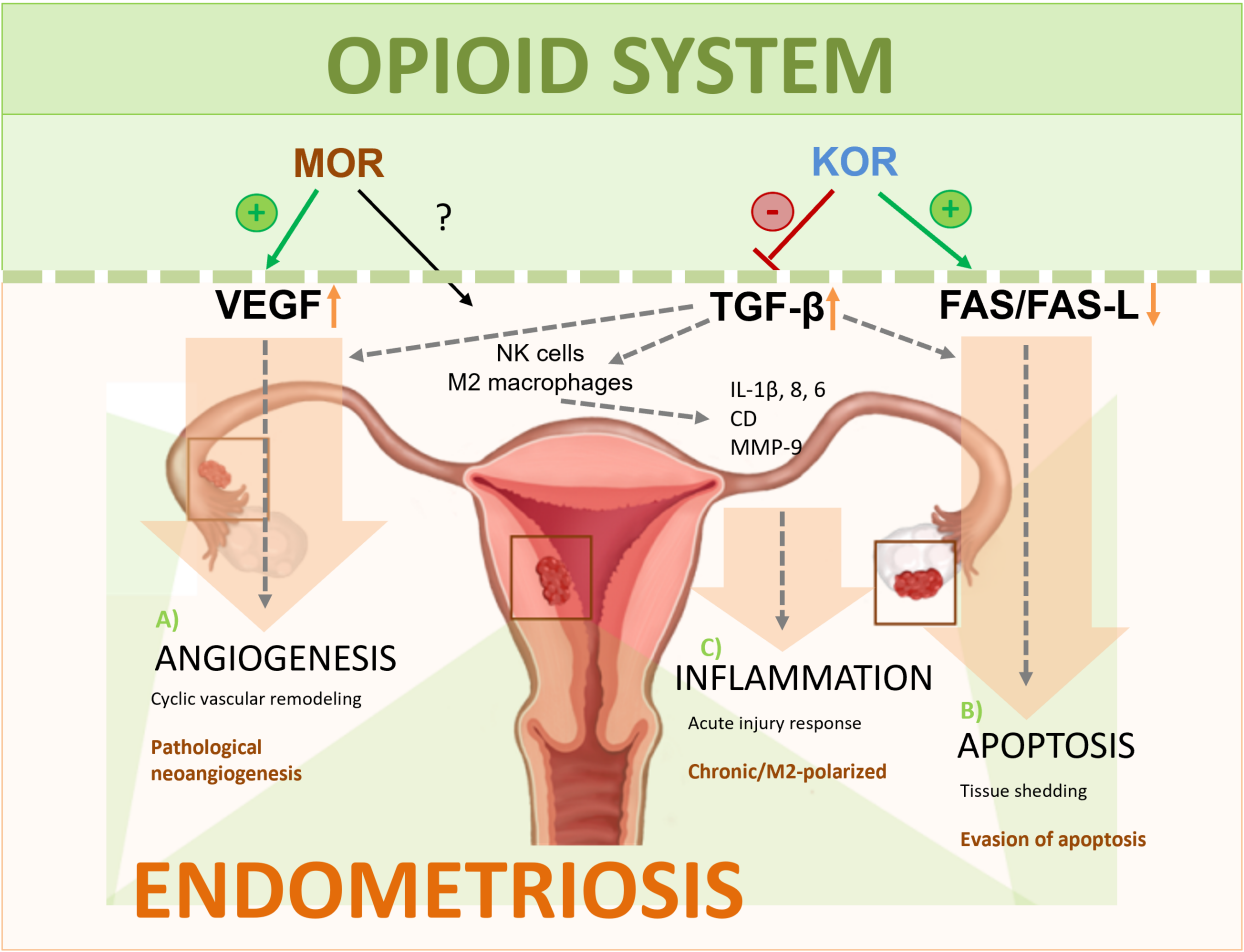
The mechanistic interplay between the opioid system and the hallmarks of endometriosis. The progression of the disease is characterized by dysregulation of three core biological processes: **(A)** angiogenesis, **(B)** apoptotic resistance, and **(C)** chronic inflammation. The orange arrows and labels depict the effects of endometriosis. The green panel illustrates the mechanistic role of the opioid system within these key processes. While direct clinical evidence is still emerging, the opioid system acts as a putative regulator; specifically, Mu-opioid receptor (MOR) signaling is implicated in enhanced VEGF expression and invasion, whereas Kappa-opioid receptor (KOR) activation serves as a homeostatic regulator by inhibiting TGF- β pathways and restoring apoptotic sensitivity. Note: This schematic focuses on localized cellular and molecular pathways; systemic hormonal regulations are excluded for clarity.

Regarding apoptosis, it has been shown that different opioid concentrations induce differential actions in this process (68); however, KOR is a known inducer of apoptosis via the FAS/FAS-L pathway in human endometrial cells (48). Moreover, an inhibitory effect of KOR on TGF-β1 production from these cells has been suggested (69). In endometriosis, the levels of TGF-β are upregulated (12), causing a decrease in the apoptotic response of cells. In this regard, it has been observed that KOR downregulation could cause a decrease in apoptosis, both via non-activation of the FAS/FASL pathway and non-inhibition of TGF-β (48) (**Figure 1**).

Nevertheless, many contradictory data have been found regarding the role of opioids in mitogenic activity. While opioids decrease cell proliferation and increase apoptosis in various cell systems, there are several cases, mainly concerning immune or nervous system cells, in which growth is stimulated by opioids (70). Studies on lung cancer cells have shown that opioid analgesics, such as morphine, which are MOR agonists, may inadvertently promote cancer progression when used for analgesia. Morphine-induced phosphorylation of the EGF receptor (EGFR) occurs via opioid receptors, leading to downstream MAPK/ERK and Akt phosphorylation, cell proliferation, and increased invasion (71). In another study, chronic exposure to morphine was found to stimulate mitogenic signaling via co-activation of Platelet-Derived Growth Factor (PDGF)-β receptors in the kidneys of control and sickle cell mice (72). Conversely, matrix MMPs play a crucial role in tissue invasion, and opioids have been shown to attenuate MMP-2 and -9 secretion and mRNA levels in the MCF-7 breast cancer cell line (73).

### 6.3 Opioid signaling and infertility

As previously discussed, infertility is a hallmark symptom of endometriosis. The clinical relevance of this association is underscored by the fact that 25–50% of infertile women are diagnosed with endometriosis, while 30–50% of women with the disease suffer from infertility. Compelling evidence also links the opioid system to these reproductive challenges. Hormonal dysregulation represents a primary underlying cause; specifically, Endomorphin-1 (EM-1) and MOR appear to modulate the hypothalamic-pituitary-ovarian (HPO) axis. In this context, MOR antagonists, such as naloxone, have been shown to restore reproductive hormone levels in murine models (74). Additionally, β-endorphin has been shown to increase the concentration of estrogen receptors in the endometrium, possibly fostering a hyperestrogenic state (46). This mechanism aligns with the estrogen upregulation, which is characteristic of endometriosis, suggesting a potential link between opioid signaling and the hyperestrogenic environment that drives disease progression. Beyond endocrine factors, opioids influence folliculogenesis and chronic inflammation, both of which are critical determinants of reduced fertility (25).

## 7 The therapeutic paradox: pain vs. pathogenesis

In terms of endometriosis-associated pain, opioids have been widely studied (22). Although they do not represent standard therapy, they are often used to treat related abdominal and pelvic pain (75). However, the conclusions of our review align with the evidence synthesized by Guan et al. (2023) (22), suggesting that current treatment options are frequently ineffective and that the adverse effects of opioid drugs on reproduction may be more significant than previously realized. This inefficacy may be partly explained by the fact that estrogen plays a significant role in modulating endogenous opioid neurotransmission and associated psychophysical responses to pain stressors (76). Taken together, this collective evidence underscores the critical need for research into novel therapeutic pathways that target pain without compromising fertility (22). Additionally, the well-known risks of addiction and dependence associated with traditional MOR agonists represent a major clinical barrier, further necessitating the search for safer alternatives.

In this context, experimental models have shown that the acute administration of KOR agonists alleviates pelvic mechanical hypersensitivity in endometriosis mice in a dose-dependent manner (77). These findings suggest that KOR modulation could be an effective approach for the chronic treatment of endometriosis-associated pain. This is particularly relevant as KOR and DOR modulation is gaining clinical importance due to the development of new therapies that avoid the side effects of conventional MOR agonist drugs (78–81). Similarly, the NOP receptor system has emerged as a promising target; Guan et al. have recently documented the expression of NOP receptors in endometriosis-associated nerve fibers, suggesting that its modulation could offer a novel therapeutic avenue to manage neurogenic pain while potentially reducing the adverse reproductive effects associated with traditional opioid signaling (22). Notably, the NOP system can act as a functional anti-opioid modulator, offering the prospect of mitigating drug-seeking behavior and polydrug addiction.

Given these considerations, understanding how the opioid system could be actively involved in these processes during the development of endometriosis, specifically its role in inflammation, angiogenesis, and the evasion of apoptosis, is essential for identifying new therapeutic targets. Conversely, by using opioids solely for pain management, we could unknowingly alter the mechanisms involved in the pathology, possibly exacerbating it, and, therefore, compromising the patient’s fertility. Moreover, some studies have shown sex-specific effects of opioid analgesic mechanisms (82), which could further complicate the acquisition of disease-specific knowledge, yet make this research all the more necessary. In the pursuit of precision medicine, the emerging role of micro-RNAs (miRNAs) could be crucial. As highlighted in recent systematic reviews on liquid biopsy (83), these epigenetic regulators may offer a window into the molecular state of the disease; specifically, they could serve to evaluate the baseline dysregulation of opioid pathways in endometriosis and, critically, to monitor how the administration of opioid drugs might further disrupt these mechanisms. Thus, miRNAs could function as non-invasive biomarkers to assess both pain signaling and the potential adverse effects of therapeutic intervention in these patients.

The perspective provided in this review represents a novel effort to bridge the gap between neurobiology and reproductive medicine, offering a comprehensive framework for non-hormonal therapeutic targets. However, it is important to recognize that while the mechanistic evidence regarding NOP and KOR modulation is compelling, much of it currently relies on experimental animal models. The inherent physiological differences between species and the clinical heterogeneity of endometriosis present challenges for the direct translation of these findings. Acknowledging these gaps is crucial, as they define the boundaries of our current understanding while highlighting the path forward for clinical validation.

## 8 Conclusion and future perspectives

Endometriosis remains a complex reproductive disorder. Diagnostic delays and limited therapeutic options frequently compromise patients’ quality of life and fertility. The impact of this disease goes beyond physical symptoms, negatively affecting women’s mental health and their social and professional lives. Therefore, it is urgent to investigate the basis of endometriosis, because early diagnosis and targeted treatment can mitigate pain, prevent progression, and ultimately improve quality of life and preserve fertility.

Current pharmacological interventions, largely based on hormonal suppression, are often incompatible with pregnancy and carry significant side effects, highlighting the urgent need for non-hormonal targets (84). The opioid system emerges as a promising yet dual-natured candidate in this landscape. Our review underscores its fundamental role in endometrial physiology, including its influence on inflammation, angiogenesis, and apoptosis—processes that are systematically dysregulated in endometriosis. However, a critical “opioid paradox” exists: while these molecules offer a pathway for pain management and the possibility of modulating disease progression, their systemic and chronic use may inadvertently impair ovarian steroidogenesis, oocyte quality, and endometrial receptivity (85).

Beyond their palliative role in pain management, we propose that opioids are putative modulators of the underlying mechanisms that drive endometriosis-associated subfertility. In this regard, miRNAs may offer new insights into the modulation of opioid pathways, representing a promising avenue for the future exploration of non-invasive biomarkers to assess pain signaling and therapeutic response.

Furthermore, the feasibility of prospective clinical analyses on human subjects should be prioritized to evaluate the role of the opioid system within the landscape of infertility and ARTs. Investigating how opioid signaling correlates with oocyte quality and embryo implantation rates could determine whether site-specific modulation can improve both pain management and pregnancy outcomes.

We hypothesize that a targeted approach to the opioid system could provide a novel dual-action framework: serving as a diagnostic biomarker for impaired endometrial receptivity and as a non-hormonal therapeutic strategy to restore ovulatory and menstrual regularity. It is imperative to transition from systemic analgesia to site-specific modulation to ensure that managing chronic pain does not compromise the reproductive capacity of these patients. Future research should prioritize the functional characterization of opioid receptors within the endometriotic microenvironment to transform them into precision tools for fertility preservation.
